# Sleep Health Ambassadors in Greater Detroit: A Model for Religio-Culturally Conscious Care in Places of Worship From Dearborn to Hamtramck

**DOI:** 10.7759/cureus.59890

**Published:** 2024-05-08

**Authors:** Bilal Irfan, Aneela Yaqoob

**Affiliations:** 1 Michigan Alzheimer's Disease Center, University of Michigan, Ann Arbor, USA; 2 Center for Bioethics, Harvard Medical School, Boston, USA; 3 Infectious Diseases, Beaumont Hospital, Taylor, USA

**Keywords:** religiously conscious care, disparities, health ambassadors, sleep health, culturally competent care

## Abstract

An innovative healthcare delivery model in Greater Detroit is proposed to integrate religious and cultural identities with health strategies to address specific disparities, such as higher rates of diabetes and cardiovascular diseases linked to poor sleep, among minority communities, particularly among its diverse Muslim population. This model advocates for culturally conscious care, deeply appreciating the sociocultural determinants of health. It proposes utilizing mosques as community hubs to deploy sleep health ambassadors trained in sleep science and cultural sensitivity. These ambassadors would engage the community through trusted platforms, offering tailored health interventions aligned with religious practices and cultural norms. This approach not only promises improved health outcomes, such as enhancements in sleep quality, reductions in sleep-related health issues, and increased community health awareness, but also empowers the community by incorporating local religious leaders and stakeholders in program planning and implementation, for example, through the introduction of tailored sleep hygiene workshops that align with the timing of religious practices, such as Ramadan, and culturally sensitive screening for sleep apnea. Success will be measured by improvements in self-reported sleep quality, a reduction in daytime sleepiness, and community surveys assessing awareness and engagement. By demonstrating efficacy in managing sleep health, this model could scale to address broader health issues, ensuring interventions are culturally appropriate and effectively managed within community-specific contexts. This model holds the promise of significantly reducing health disparities by adapting health interventions to the cultural and religious contexts of communities, potentially transforming the landscape of community health management.

## Editorial

In the heart of Greater Detroit, an innovative model of healthcare delivery has the potential to take root, one that intertwines the intricacies of religious and cultural identity with the pursuit of enhanced community health outcomes. Particularly within the Muslim community, which forms a significant segment of Detroit's demographic mosaic, alongside substantial Black and Arab American populations, many of which maintain overlapping ethnic identities with an Islamic religious affiliation, there is a vivid interplay of belief, tradition, and need that calls for a bespoke approach to healthcare. This is especially pertinent in the realm of sleep health, an often-overlooked aspect of well-being but crucial for overall health.

The concept of culturally conscious care emerges as a crucial framework for addressing the unique health disparities faced by these communities. Culturally conscious care respects and integrates the cultural beliefs and needs of individuals into their healthcare. This approach is not merely about language or superficial understanding but involves a deep, nuanced appreciation of the sociocultural determinants that influence health behaviors and outcomes. In Detroit, where the intersection of ethnicity, religion, and health inequities presents a complex challenge, integrating culturally conscious care in sleep health management could serve as a beacon for broader healthcare strategies [1].

Sleep disorders and poor sleep quality have been linked to numerous health issues, including cardiovascular disease, diabetes, and mental health disorders, which disproportionately affect minority communities [2]. Tailored health interventions that consider cultural and religious norms can significantly enhance the receptivity and effectiveness of health programs. For instance, the timing of sleep education and intervention programs in Muslim communities could consider prayer times, dietary practices during Ramadan, and other cultural practices to increase engagement [3].

The role of places of worship, particularly mosques, in the Muslim community offers a unique platform for health interventions. These are not just places for spiritual gatherings but serve as community hubs where trust and camaraderie are intrinsic. The proposal to introduce sleep health ambassadors in these settings is a novel approach that leverages this trust. These ambassadors, trained in the basics of sleep science and culturally conscious care, can act as liaisons between health professionals and the community, offering guidance, education, and resources tailored to the community’s needs [4]. Local imams will be pivotal in endorsing and advocating for the program, while health professionals will provide medical oversight and training for the sleep health ambassadors.

Such ambassadors could be utilized to organize workshops after Friday prayers or coordinate with local health professionals to provide screenings for sleep disorders. These programs could be tailored to address the specific sleep health challenges identified within the community, such as those arising from lifestyle, dietary habits, or occupational stresses that may be prevalent. Additionally, by using culturally relevant communication strategies and materials, these ambassadors can effectively disseminate information and raise awareness about the importance of good sleep hygiene.

The potential effectiveness of this model lies in its community-centric approach. By involving local religious leaders, imams, and community stakeholders in the planning and implementation phases, the programs ensure greater relevance and acceptance [5]. Furthermore, such a strategy not only aids in the effective management of sleep health but also empowers the community by providing them with the tools and knowledge to take charge of their health. This empowerment is crucial in minority communities where there is often a historical distrust of mainstream medical systems.

Furthermore, the integration of sleep health into culturally conscious care in Detroit could serve as a scalable model for other health interventions. Demonstrating success in this area could pave the way for addressing other health issues within the community through similar culturally tailored frameworks. This could include mental health, chronic disease management, and preventive care, all of which are crucial areas of health that require community-specific strategies to effectively manage.

However, the challenges of implementing such a program should not be underestimated. These include ensuring the sustained funding of health programs, training ambassadors to a high standard, and continuously engaging the community without overwhelming the cultural and religious essence of places of worship. Moreover, the evaluation of such programs must be thoughtfully designed to capture not only the health outcomes but also the acceptability and cultural sensitivity of the interventions. This could include assessments on a bi-annual basis of the outcomes presented, regular focus groups with community members, and tracking of participation rates in health workshops, to name a few.

Despite the potential of this model, its implementation is not without challenges. Financial constraints, logistical complexities, and cultural sensitivities present significant barriers to the deployment of sleep health ambassadors in mosques. Financially, securing sustained funding is crucial, as inconsistent funding can lead to interruptions that may erode trust within the community. Logistically, the recruitment, training, and retention of culturally competent health ambassadors require careful planning and resources. Culturally, while the model leverages religious and community structures, it must also navigate the diverse interpretations and practices within Islam that might affect health-seeking behavior.

Strategically overcoming these challenges involves a multifaceted approach. Financial sustainability could be approached through partnerships with local health organizations, grant funding, and community fundraising, which not only provide financial resources but also enhance community ownership of the project. Logistically, partnerships with local educational institutions can provide a steady pipeline of trained health ambassadors. To address cultural challenges, the program should include continuous dialogue with community leaders and members to adapt the interventions to align with evolving community norms and values. These strategies emphasize the importance of flexibility and community involvement in the model's success, underscoring a key take-home message: community-specific, culturally conscious health interventions can significantly improve health outcomes if they are adaptable, adequately supported, and collaboratively implemented.

The argument for addressing sleep health disparities through culturally conscious care is further supported by empirical evidence. Studies have shown that culturally tailored health interventions increase participation and compliance rates among minority groups. Nevertheless, the success of such culturally integrated health programs heavily depends on the extent of community buy-in and the continuous engagement of local leaders. Without strong, sustained advocacy from within the community, the impact of these programs might be limited. Additionally, the specificity of the interventions to the Detroit Muslim community might not be directly transferable to other communities or regions without significant modifications. Recognizing these limitations not only provides a more balanced view but also sets realistic expectations for stakeholders, enhancing the editorial’s credibility and robustness.

An initiative to establish sleep health ambassadors in the mosques of the Greater Detroit area offers a promising path toward improved health outcomes through culturally conscious care. By respecting and integrating the religious and cultural dimensions of the community, this proposed model acknowledges a spectrum of factors that influence health. It stands as a testament to the potential of community-specific health initiatives that honor the diverse nature of human culture, paving the way for a healthier, more inclusive future.
